# Angiographic features and progression risk factors in children with asymptomatic moyamoya disease

**DOI:** 10.3389/fneur.2024.1484132

**Published:** 2024-11-15

**Authors:** Jiyuan Wang, Qian Zhang, Fei Di, Dong Zhang

**Affiliations:** ^1^Department of Neurosurgery, Beijing Tiantan Hospital, Capital Medical University, Beijing, China; ^2^Department of Neurosurgery, Capital Institute of Pediatrics, Beijing, China; ^3^Department of Neurosurgery, Beijing Hospital, National Center of Gerontology, Beijing, China; ^4^Institute of Geriatric Medicine, Chinese Academy of Medical Sciences, Beijing, China

**Keywords:** moyamoya disease, angiography, hemorrhage, ischemia, stroke

## Abstract

**Objective:**

This study aims to explore the potential contribution of angiographic characteristics in the increased stroke risk among pediatric patients.

**Methods:**

This study retrospectively enrolled pediatric patients with ischemic, hemorrhagic, and asymptomatic moyamoya disease. Their hemispheres were categorized into five groups for the analysis of angiographic characteristics, which included Suzuki’s stage, moyamoya vessels, lenticulostriate artery, thalamotuberal artery, thalamoperforating artery, anterior choroidal arteries, posterior choroidal arteries, and posterior cerebral artery involvement.

**Results:**

Two hundred and nineteen pediatric patients with four hundred and thirty-eight hemispheres were enrolled. There was no significant difference in collateral dilatation between asymptomatic and hemorrhagic hemispheres. However, asymptomatic hemispheres had significantly lower incidence of posterior cerebral artery involvement and Suzuki’s stage compared to hemorrhagic (*p* = 0.008, *p* = 0.004) and ischemic hemispheres (*p* = 0.026, *p* < 0.001). Multivariate analysis revealed that Suzuki’s stage (*p* = 0.002, 95% CI 1.261–2.867) is a risk factor for ischemia, while age (*p* < 0.001, 95% CI 0.712–2.014) and posterior cerebral artery involvement (*p* = 0.037, 95% CI 0.087–13.377) are risk factors for hemorrhage.

**Interpretation:**

Angiographic features in children with asymptomatic moyamoya disease resemble those observed in the hemorrhagic pediatric patients, indicating that greater attention should be focused on the risk of future hemorrhage in these patients, rather than on ischemia. Additionally, studies have demonstrated a correlation between posterior cerebral artery involvement and age with the incidence of hemorrhage. Monitoring angiographic characteristics may assist in tracking the transition from asymptomatic to symptomatic hemispheres.

## Introduction

Moyamoya disease (MMD) is characterized by chronic progressive stenosis of the terminal internal carotid artery and an abnormal vascular network at the base of the skull (1). The exact cause of this distinctive cerebrovascular condition remains unknown. Predominantly observed in East Asia, patients with MMD present with various clinical symptoms, including ischemia, hemorrhage, and epilepsy (2). The prevalence of asymptomatic MMD patients is increasing due to the widespread use of non-invasive imaging techniques, such as magnetic resonance angiography (MRA) (3). Previous studies have suggested that asymptomatic MMD is not a stable condition, and carries a risk of progressing to symptomatic manifestations, such as ischemia or hemorrhage (3, 4). A historical prospective cohort study in Japan revealed that asymptomatic patients managed conservatively faced an annual stroke risk of 3.2% (3).

The current effective treatment for MMD is cerebral revascularization, including extra-intracranial bypass surgery, indirect revascularizations, and combined revascularization (5). These interventions have shown benefits for symptomatic patients (6, 7). However, there is a lack of clear management options for asymptomatic patients. A study revealed that surgery did not effectively reduce the incidence of stroke progression in North American asymptomatic patients over a 5-year follow-up period (8). This highlights the necessity for a more precise characterization of asymptomatic patients to guide management strategies.

Several recent studies have proposed imaging risk factors for ischemia or hemorrhage in asymptomatic MMD (4, 9, 10). However, the majority of these studies have focused on adult patients, leaving a gap in research regarding pediatric patients, who represent another peak age group for the onset of MMD (11). This study aims to explore the potential contribution of angiographic variations in the increased stroke risk among pediatric patients.

## Methods

### Participants and settings

We retrospectively collected data on patients diagnosed with moyamoya disease who were consecutively admitted to Beijing Tiantan Hospital Affiliated to Capital Medical University from January 2019 to December 2023.

Enrolled patients in this study met specific criteria: (1) diagnosed with MMD by digital subtraction angiography (DSA) according to the 2021 guidelines (12); (2) aged under 18 years; (3) presented with asymptomatic manifestations, hemorrhagic events, or ischemic patterns, including cerebral infarction and transient ischemic attack (TIA); (4) had not undergone previous cerebral revascularization surgery. Patients diagnosed with unilateral, quasi-moyamoya disease or moyamoya syndrome were excluded from the study. Additionally, patients were excluded if they lacked sufficient angiographic or clinical data, or if they had primary epilepsy, congenital aphasia, or movement disorders. Asymptomatic MMD refers to patients who have not experienced TIA, ischemic stroke, hemorrhagic stroke, seizures or any other neurological deficits (4). These patients are typically identified due to unexplained dizziness, headaches, or incidental findings during physical examination. Two neurosurgeons independently reviewed and evaluated the cases, with any discrepancies resolved through thorough discussion in a roundtable meeting. This study was approved by the Ethics Committee of Beijing Tiantan Hospital Affiliated to Capital Medical University (KY2022-051).

Patients were classified into three groups based on their clinical presentations: hemorrhagic group, ischemic group, and asymptomatic group. The study included hemispheres from these patients, which were further divided into five subgroups based on the patient’s origin and location of symptoms. For instance, in patients with hemorrhagic MMD, the hemispheres were categorized into two groups: those with hemorrhages (hh-hemispheres) and those who are asymptomatic (ah-hemispheres). Similarly, the hemispheres with symptoms in patients with ischemic symptoms (including cerebral infarction and TIA) were designated as ii-hemispheres, while asymptomatic hemispheres were labeled as ai-hemispheres. The hemispheres of asymptomatic patients were identified as aa-hemispheres. The localization of hemorrhagic and ischemic hemispheres was determined based on clinical manifestations and imaging.

### Angiographic evaluation

**Figure 1:**
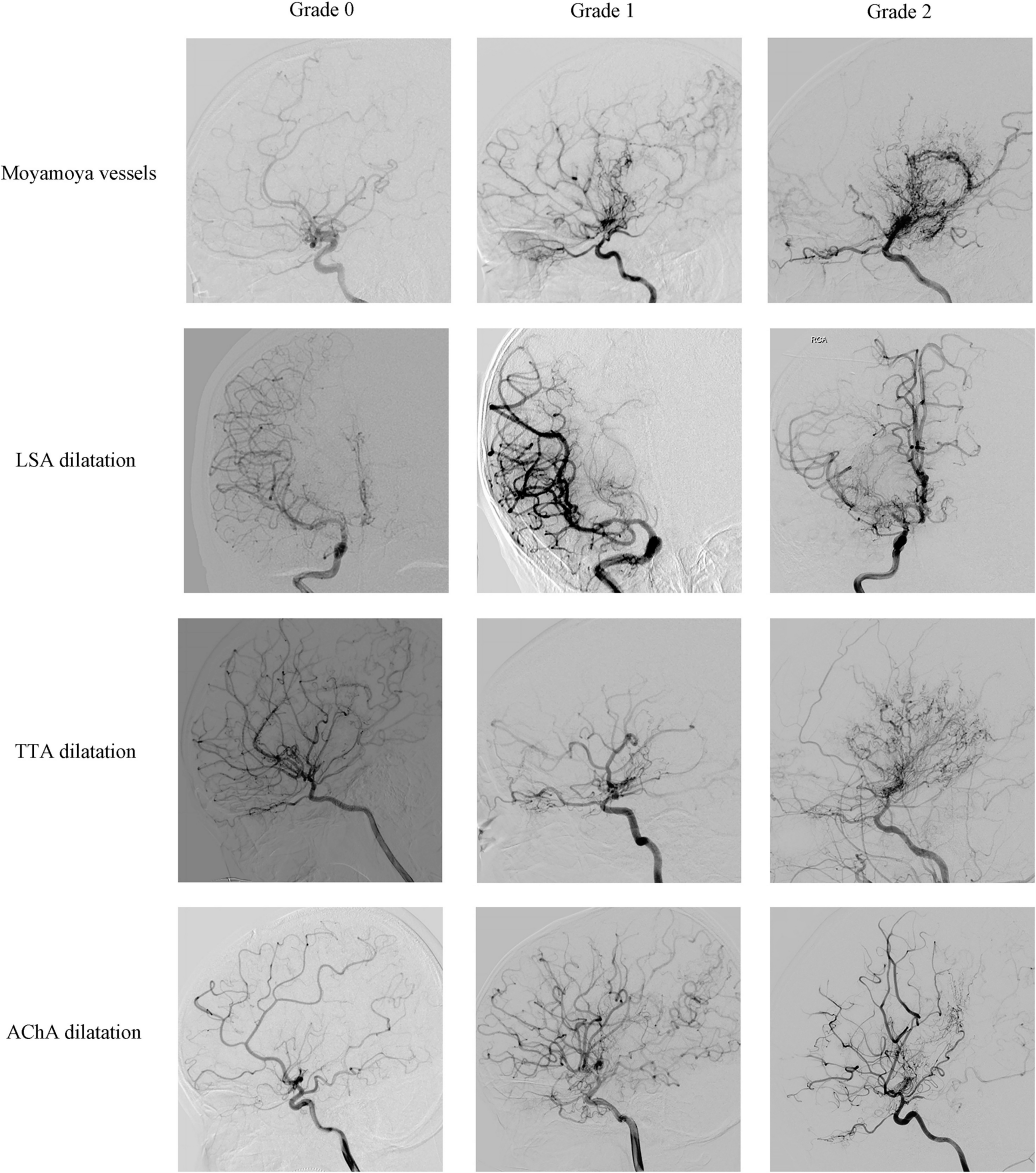
Representative cerebral angiography of the grading of moyamoya vessels, LSA dilatation, TTA dilatation and AChA dilatation. Moyamoya vessels were categorized as absent (grade 0), sparse (grade 1), or dense (grade 2); LSA dilatation, TTA dilatation and AChA dilatation were categorized as normal (grade 0), dilation with distal branches (grade 1), or dilatation with abnormal branch extension to provide blood supply to other areas (grade 2).

**Figure 2:**
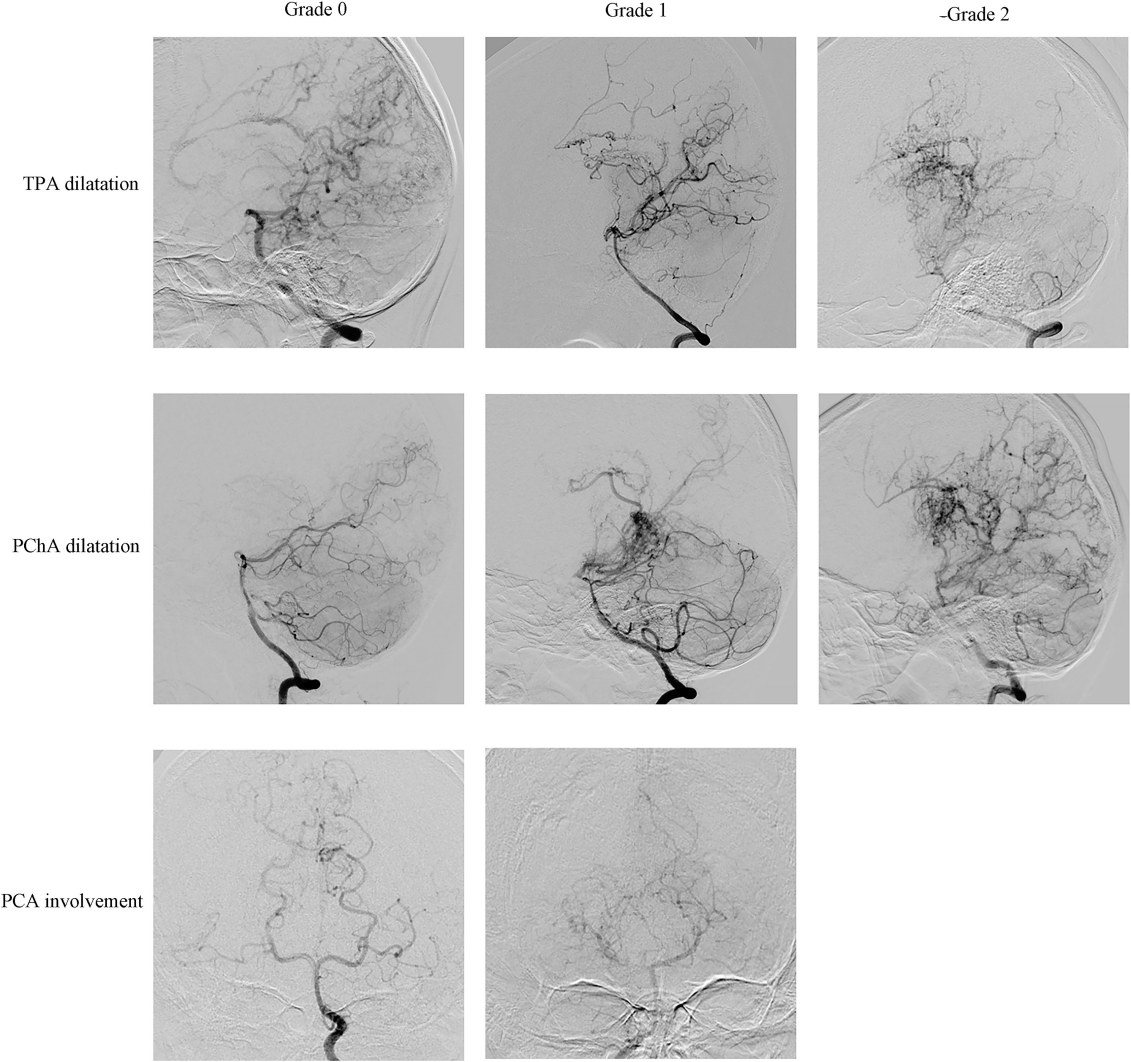
Representative cerebral angiography of the grading of TPA dilatation, PChA dilatation and PCA involvement. TPA dilatation and PChA dilatation were categorized as normal (grade 0), dilation with distal branches (grade 1), or dilatation with abnormal branch extension to provide blood supply to other areas (grade 2); PCA involvement was categorized as normal (grade 0), stenosis or occlusion (grade 1).

Preoperative DSA of the included hemispheres was retrospectively analyzed by an image interpretation team consisting of two neurosurgeons with extensive experience in neuroimaging interpretation. The interpretation involved an assessment of Suzuki’s stage, moyamoya vessels, lenticulostriate artery (LSA), thalamotuberal artery (TTA), thalamoperforating artery (TPA), anterior choroidal arteries (AChA), posterior choroidal arteries (PChA) and posterior cerebral artery (PCA). Each hemisphere was classified into six stages according to Suzuki’s angiographic staging (13). Moyamoya vessels were categorized as absent (grade 0), sparse (grade 1), or dense (grade 2) (14, 15). The assessment of collateral vessels was conducted using the grading system established in supplementary studies of the Japanese Moyamoya Disease in Adults (JAM) trial (9, 16, 17), where grade 0 indicated normal vessels, grade 1 represented dilation with distal branches, and grade 2 represents dilatation with abnormal branch extension to provide blood supply to other areas, considered positive for dilation (Figures 1, 2). The extent of involvement of PCA was categorized into grade 0 for normal and grade 1 for stenosis or occlusion (Figure 2).

### Statistical analysis

All statistical analyses were conducted using SPSS version 26.0 (SPSS Inc., Chicago, IL, United States). For continuous variables, either Student’s *t*-test or the Mann–Whitney *U* test was employed, while the chi-square test or Fisher’s exact test was utilized for categorical variables to compare baseline differences. Single-factor and multi-factor logistic regression models were used to identify risk factors. A significance level of *p* < 0.05 was set for the two-tailed tests.

## Results

### Demographic and history information

**Figure 3:**
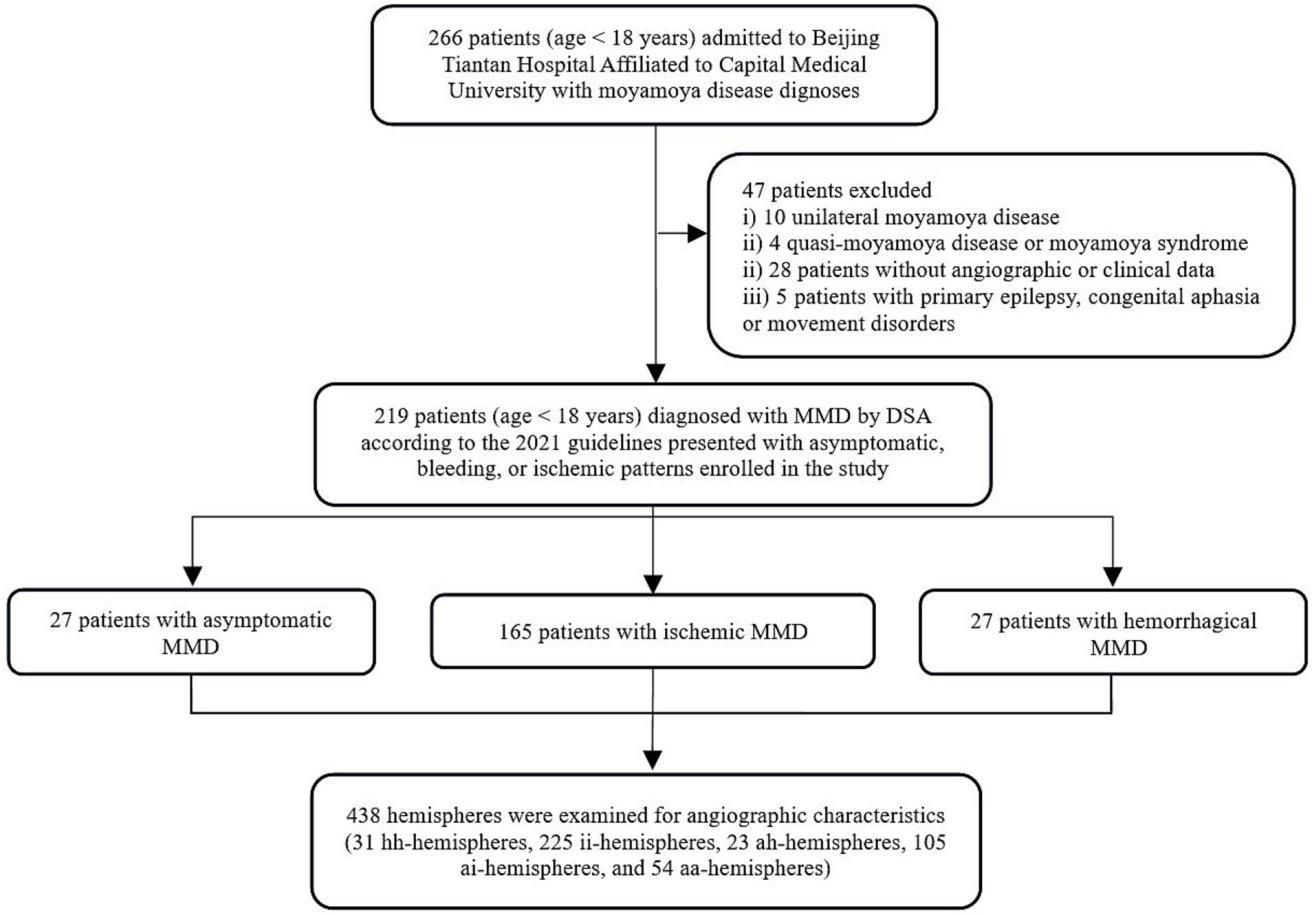
Workflow illustration diagram for study inclusion/exclusion process. MMD, moyamoya disease; DSA, digital subtraction angiography.

This retrospective study analyzed a total of 219 hospitalized patients, comprising 438 cerebral hemispheres. The cohort consisted of 204 consecutive cases from a five-year period and 15 individuals from a previous database, with 4 being asymptomatic and 11 experienced hemorrhages. The detailed inclusion and exclusion process is illustrated in the workflow diagram in Figure 3. Among 219 patients with MMD analyzed, 27 belonged to hemorrhagic group, 165 to the ischemic group, and 27 to the asymptomatic group. Demographic information showed that patients in the asymptomatic and ischemic groups were significantly younger compared to patients in hemorrhagic group (*p* < 0.001). There was no significant difference in sex distribution among the groups (Table 1). Regarding hemispheres, out of the 438 included, there were 31 hh-hemispheres, 225 ii-hemispheres, 23 ah-hemispheres, 105 ai-hemispheres, and 54 aa-hemispheres examined for angiographic characteristics.

**Table 1:** Comparison of baseline characteristics of study cases.

	Asymptomatic group	Ischemic group	Hemorrhagic group	P1	P2	P3
No. of patients (hemispheres)	27 (54)	165 (330)	27 (54)			
Age (mean ± SD), IQR	9.78 ± 3.20 (8.0–12.0)	9.75 ± 3.31 (7.0–12.0)	13.52 ± 3.48 (12.5–16.0)	0.897	<0.001^*^	<0.001^*^
Female (%)	51.85%	55.76%	51.85%	0.835	1	0.835

P1 value indicates asymptomatic group vs. ischemic group; P2 value indicates asymptomatic group vs. hemorrhagic group; P3 value indicates ischemic group vs. hemorrhagic group. ^*^p < 0.05.

### Angiographic feature assessment

The baseline angiographic characteristics of aa-hemispheres group, ii-hemispheres group, and hh-hemispheres group were compared in pairs, including Suzuki’s stage, moyamoya vessels, LSA dilation, TTA dilation, TPA dilation, AChA dilation, PChA dilation, and PCA involvement (Table 2). The positive rates of LSA (*p* = 0.040) and AChA (*p* = 0.024) in aa-hemispheres group were significantly higher than those in ii-hemispheres group, while TTA (*p* = 0.100), TPA (*p* = 0.619) and PChA (*p* = 0.280) exhibited no significant differences. However, the five collateral assessment results of aa-hemispheres group did not show significant differences compared to those of hh-hemispheres group. Additionally, the incidence of PCA involvement in aa-hemispheres group was notably lower compared to ii-hemispheres group and hh-hemispheres group (*p* = 0.026, *p* = 0.008). Multivariate analysis was conducted separately on aa-hemispheres group, ii-hemispheres group, and hh-hemispheres group, revealing one factor associated with infarction: Suzuki’s stage (*p* = 0.002, 95% CI 1.261–2.867). In addition, two factors were linked to hemorrhage: age (*p* < 0.001, 95% CI 0.712–2.014) and PCA involvement (*p* = 0.037, 95% CI 0.087–13.377) (Tables 3, 4).

**Table 2:** Comparison of angiographic characteristics of hemispheres.

	aa-hemispheres group	ii-hemispheres group	hh-hemispheres group	P1	P2	P3
Suzuki’s stage				<0.001^*^	0.004^*^	0.252
1	0 (0.0%)	7 (3.0%)	1 (3.2%)			
2	24 (44.4%)	11 (4.9%)	1 (3.2%)			
3	13 (24.1%)	105 (46.7%)	11 (35.5%)			
4	14 (25.9%)	85 (37.8%)	18 (58.1%)			
5	2 (3.70%)	15 (6.7%)	0 (0.0%)			
6	1 (1.85%)	2 (0.9%)	0 (0.0%)			
Moyamoya vessels				0.283	0.179	0.179
0	9 (16.7%)	28 (12.4%)	1 (3.2%)			
1	26 (48.1%)	102 (45.3%)	14 (45.2%)			
2	19 (35.2%)	95 (42.2%)	16 (51.6%)			
LSA				0.040^*^	0.582	0.006^*^
0	7 (13.0%)	84 (37.3%)	3 (9.7%)			
1	37 (68.5%)	123 (54.7%)	20 (64.5%)			
2 (positive)	10 (18.5%)	18 (8.0%)	8 (25.8%)			
TTA				0.100	0.762	0.035^*^
0	11 (20.4%)	81 (36.0%)	7 (22.6%)			
1	35 (64.8%)	128 (56.9%)	18 (58.1%)			
2 (positive)	8 (14.8%)	16 (7.1%)	6 (19.4%)			
TPA				0.619	0.471	0.770
0	21 (38.9%)	122 (54.2%)	16 (51.6%)			
1	29 (53.7%)	78 (34.7%)	11 (35.5%)			
2 (positive)	4 (7.4%)	25 (11.1%)	4 (12.9%)			
AChA				0.024^*^	0.503	0.005^*^
0	12 (22.2%)	12 (22.2%)	4 (12.9%)			
1	21 (38.9%)	21 (38.9%)	11 (35.5%)			
2 (positive)	21 (38.9%)	21 (38.9%)	16 (51.6%)			
PChA				0.280	0.779	0.167
0	22 (40.7%)	150 (66.7%)	20 (64.5%)			
1	22 (40.7%)	46 (20.4%)	4 (12.9%)			
2 (positive)	10 (18.5%)	29 (12.9%)	7 (22.6%)			
PCA involvement	12 (22.2%)	88 (39.1%)	16 (51.6%)	0.026^*^	0.008^*^	0.242

LSA, lenticulostriate artery; TTA, thalamotuberal artery; TPA, thalamoperforating artery; AChA, anterior choroidal artery; PChA, posterior choroidal arteries; PCA, posterior cerebral artery; P1 value indicates aa-hemispheres group vs. ii-hemispheres group; P2 value indicates aa-hemispheres group vs. hh-hemispheres group; P3 value indicates ii-hemispheres group vs. hh-hemispheres group. ^*^p < 0.05.

**Table 3:** Multivariate analysis of ischemia in hemispheres of pediatric patients with asymptomatic moyamoya disease.

	Coef	*p*	OR	95% CI	
Age	−0.010	0.846	0.990	0.899	1.091
Sex	−0.330	0.319	0.719	0.376	1.376
Suzuki’s stage	0.642	0.002^*^	1.900	1.261	2.867
LSA	0.812	0.080	2.251	0.908	5.601
AChA	0.681	0.052	1.975	0.994	3.931
PCA involvement	0.185	0.650	1.203	0.541	2.667
const	−0.955				

LSA, lenticulostriate artery; AChA, anterior choroidal artery; PCA, posterior cerebral artery; Coef, coefficient; OR, odds ratio. ^*^p < 0.05.

**Table 4:** Multivariate analysis of hemorrhage in hemispheres of pediatric patients with asymptomatic moyamoya disease.

	Coef	*p*	OR	95% CI	
Age	0.350	<0.001^*^	1.419	0.712	2.014
Sex	0.707	0.220	2.029	0.105	4.118
Suzuki’s stage	0.228	0.452	1.256	0.306	1.576
PCA involvement	1.296	0.037^*^	3.656	0.087	13.377
const	−6.035				

PCA, posterior cerebral artery; Coef, coefficient; OR, odds ratio. ^*^p < 0.05.

The angiographic characteristics of aa-hemispheres group, ai/ah-hemispheres group, and ii/hh hemispheres group were further compared. The incidence of PCA involvement of aa-hemispheres group was significantly lower than ah-hemispheres group (*p* = 0.032). Additionally, Suzuki’s stage of ai-hemispheres group was significantly lower than that of ii-hemispheres group (*p* < 0.001). There were no significant differences in collateral dilation between ai-hemispheres group and ii-hemispheres group, as well as between ah-hemispheres group and hh-hemispheres group. More details can be found in Supplementary Table S1.

## Discussion

Our study found that age and PCA involvement are two significant risk factors for hemorrhage in children with asymptomatic MMD. Previous research has shown that adults are more likely to experience hemorrhagic symptoms than children, suggesting a relationship between age and hemorrhage. However, this correlation had not been established in pediatric patients until our study. We observed that the children who developed hemorrhage were significantly older than those who remained asymptomatic. Age was identified as an independent predictor in the multivariate analysis, suggesting that, when assessing bleeding risk and making clinical decisions for these patients, it is essential to consider age stratification in pediatric patients rather than relying solely on staging based on imaging. Our study revealed that PCA involvement is a significant predictor of hemorrhage in children with asymptomatic MMD, consistent with previous research in adults (18). This link could be due to PCA stenosis and occlusion leading to ischemia in the posterior circulation, increasing hemodynamic stress on collateral vessels such as ChA and increasing the risk of hemorrhage. Furthermore, research suggests that PCA involvement in MMD is associated with a higher risk of negative outcomes, such as increased rates of ischemic perioperative complications, and poorer functional outcomes (19). Therefore, it is essential to focus on the evaluation of PCA involvement in MMD cases. Previous studies have suggested a relationship between ChA dilation and hemorrhage in MMD (20), a study comparing hemorrhagic and ischemic MMD in children found a significant increase in ChA dilation in the hemorrhagic group, suggesting a similar trend in pediatric patients (21). Our study produced comparable findings, indicating a significantly higher incidence of AChA dilation in the hemorrhagic group compared to the ischemic group. In 2012, Japan initiated the AMORE (Asymptomatic Moyamoya Registry) study, a multi-center prospective investigation of asymptomatic MMD. The five-year follow-up results from this study revealed that ChA dilation poses a risk for hemorrhagic stroke in adults with asymptomatic MMD (4). However, our retrospective study did not observe any difference in ChA dilation, including AChA and PChA, between hemorrhagic group and asymptomatic group. Further follow-up is needed to determine if ChA dilation can be considered a risk factor for hemorrhage in children.

In our comparison of aa-hemispheres and ii-hemispheres, we identified Suzuki’s stage as a significant risk factor, with higher Suzuki’s stages correlating with an increased risk of ischemia. This finding is consistent with the established understanding that asymptomatic MMD can be considered an early stage of symptomatic MMD. A study on the progression of asymptomatic MMD suggested that hemodynamic disturbance, rather than vascular morphological changes, play a crucial role in the transition from the asymptomatic hemisphere to the ischemic hemisphere (22). Our multivariate analysis also revealed no significant correlation between angiographic features and ischemia, except for Suzuki’s stage. However, we did observe that the dilation of certain collateral vessels (LSA, TTA, AChA) in ii-hemispheres was less pronounced compared to aa-hemispheres. This difference may be attributed to the abundant blood perfusion provided by the excessively dilated collateral vessels in asymptomatic hemispheres, preventing the manifestation of ischemic symptoms. Moving forward, future analyses should consider incorporating hemodynamic characteristics to further explore this relationship.

In addition, a comparative study between the AMORE and JAM (Japan Adult Moyamoya) cohorts indicated that there was no significant difference in LSA and ChA dilation between asymptomatic adults and those in hemorrhagic group. This finding suggests that asymptomatic adults may have a higher risk of hemorrhage (18). This observation was supported by the 5-year follow-up results of the AMORE prospective study, which revealed a higher incidence of hemorrhagic stroke in asymptomatic adults compared to ischemic stroke (4). Our study produced similar results. When comparing aa-hemispheres and hh-hemispheres, no differences were observed in the five collateral dilation studied. This indicates that asymptomatic pediatric patients may be at a greater risk of hemorrhagic stroke than ischemic stroke. This finding challenges the prevailing belief that children are more prone to ischemic events and will have significant implications for the clinical management of these patients.

**Figure 4:**
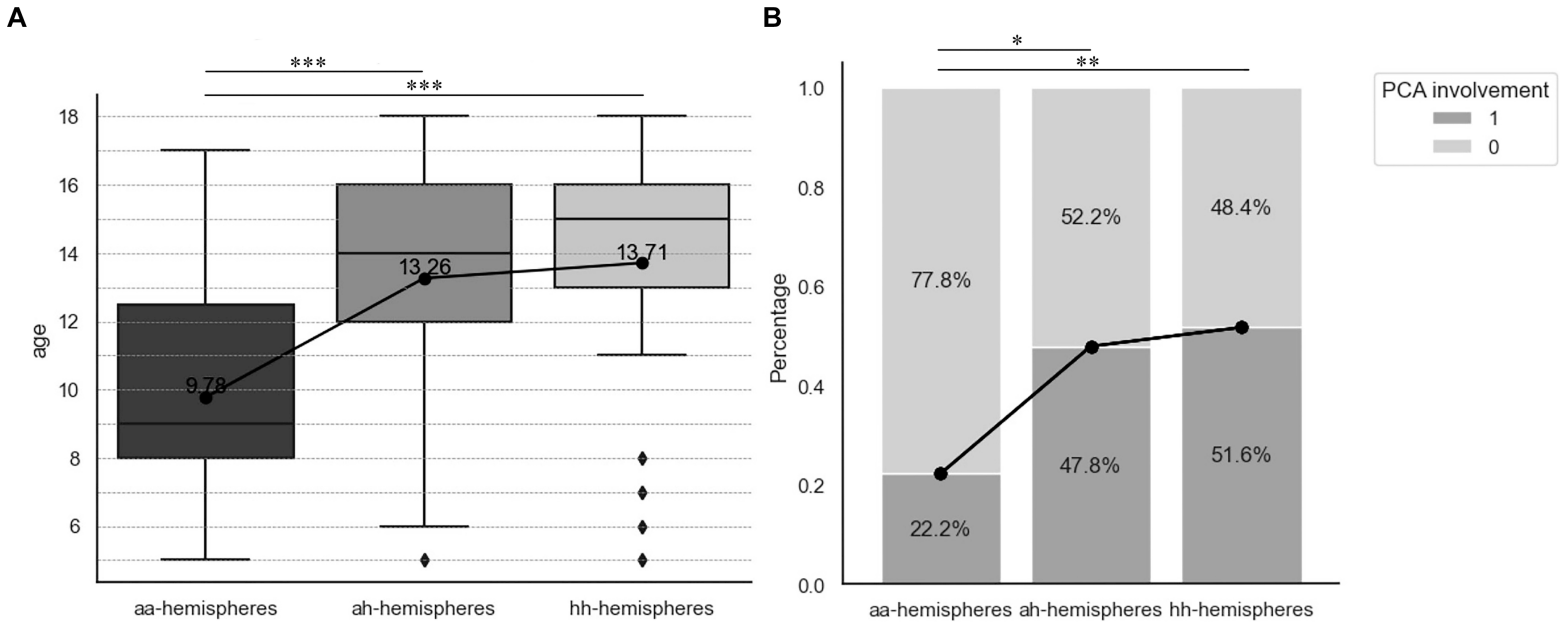
Comparison of angiographic characteristics of different hemispheres. **(A)** Box plot for the Suzuki’s angiographic stage of aa-hemispheres, aihemispheres and ii-hemispheres. **(B)** Stacked bar plot for the PCA involvement of aa-hemispheres, ah-hemispheres and hh-hemispheres. ^*^p < 0.05, ^**^p < 0.01, and ^**^p < 0.001.

Our study evaluated the asymptomatic hemispheres (ai-hemispheres and ah-hemispheres) of symptomatic patients and found that their angiographic characteristics were not similar to aa-hemispheres, but rather showed consistent characteristics with their corresponding symptomatic hemispheres. Previous research suggests that non-bleeding hemispheres in patients with hemorrhagic MMD have a tendency towards hemorrhagic stroke, with an annual risk of new hemorrhage at 2.0% (23). Therefore, it is reasonable to speculate that such hemispheres are at an increased risk of developing symptoms consistent with the contralateral hemispheres, especially for hemorrhagic stroke. Therefore early intervention in these temporarily stable hemispheres is crucial. Based on this assumption, we can consider aa-ai-ii and aa-ah-hh as hemispheres in three different stages. We further aimed to investigate the changes in risk factors across these three distinct stages. Our analysis revealed that Suzuki’s stage exhibited an increasing trend in the comparison of aa-ai-ii hemispheres. In the comparison of aa-ah-hh hemispheres, both age and the incidence of PCA involvement also demonstrated an upward trend (Figure 4). These findings suggest that the progression from asymptomatic to symptomatic hemispheres is identifiable, and the risk factors examined in our study (Suzuki’s stage, age and PCA involvement) are valuable for monitoring this progression. Previous studies have examined the hemodynamics and Suzuki’s stage of these hemispheres, concluding that the progression from aa to ah to hh is associated with changes in angiographic features, while the progression of aa to ai to ii is linked to hemodynamics (22). Our research delved into the angiographic characteristics of pediatric patients and reached similar conclusions. We further found a relationship between PCA involvement and the transition from asymptomatic to hemorrhagic pattern. Therefore, monitoring PCA is valuable, as it can assist in assessing the direction and progression of pediatric patients with asymptomatic MMD.

### Limitations

This study has several limitations. Firstly, it was a retrospective cross-sectional study with no reported follow-up results. This article is the first to systematically compare the angiographic characteristics of asymptomatic children’s hemispheres with those of children with ischemic and hemorrhagic hemispheres, highlighting the necessity for prospective studies in the future. Secondly, the sample size of this study was relatively small due to practical constraints. Future studies should aim to increase the sample size to validate these findings.

## Conclusion

Higher Suzuki’s stage correlates with ischemic events in children with MMD, while increased PCA involvement and older age are associated with hemorrhagic events. Furthermore, the angiographic characteristics of children with asymptomatic MMD more closely resembled the hemispheres with hemorrhages. It is crucial to prioritize the potential risk of progression to hemorrhage over progression to ischemia. Monitoring angiographic characteristics like Suzuki’s stage and PCA involvement can help track the transition from asymptomatic to symptomatic hemispheres. The findings of this study provide valuable insights for improving the clinical management of children with asymptomatic MMD, including refining surgical indications, determining optimal timing for surgery, and effectively monitoring patients under conservative observation.

## Data Availability

The data that support the findings of the study are available from the corresponding author upon reasonable request.
