# Supplementary material for: Angiographic features and progression risk factors in children with asymptomatic moyamoya disease
Source: Front Neurol. 2024 Nov 15;15:1484132. doi: 10.3389/fneur.2024.1484132 (PMC11604640; doi:10.3389/fneur.2024.1484132)
Supplement: Supplementary file 1 [file Table_1.docx]

Table S1. Comparison of Angiographic Characteristics of Hemispheres

|  | aa-hemispheres group | ai-hemispheres group | ii-hemispheres group | ah-hemispheres group | hh-hemispheres group | P1 | P2 | P3 | P4 |
| --- | --- | --- | --- | --- | --- | --- | --- | --- | --- |
| Suzuki’s stage |  |  |  |  |  | 0.500 | <0.001* | 0.036* | 0.829 |
| 1 | 0(0.0%) | 0(0%) | 7(3.0%) | 1(4.3%) | 1(3.2%) |  |  |  |  |
| 2 | 24(44.4%) | 35(33.3%) | 11(4.9%) | 3(13.0%) | 1(3.2%) |  |  |  |  |
| 3 | 13(24.1%) | 40(38.1%) | 105(46.7%) | 6(26.1%) | 11(35.5%) |  |  |  |  |
| 4 | 14(25.9%) | 24(22.9%) | 85(37.8%) | 12(52.2%) | 18(58.1%) |  |  |  |  |
| 5 | 2(3.70%) | 4(3.8%) | 15(6.7%) | 0(0.0%) | 0(0.0%) |  |  |  |  |
| 6 | 1(1.85%) | 2(1.9%) | 2(0.9%) | 1(4.3%) | 0(0.0%) |  |  |  |  |
| moyamoya vessels |  |  |  |  |  | 0.770 | 0.383 | 0.980 | 0.073 |
| 0 | 9(16.7%) | 21(20.0%) | 28(12.4%) | 1(4.3%) | 1(3.2%) |  |  |  |  |
| 1 | 26(48.1%) | 41(39.0%) | 102(45.3%) | 16(69.6%) | 14(45.2%) |  |  |  |  |
| 2 | 19(35.2%) | 43(41.0%) | 95(42.2%) | 6(26.1%) | 16(51.6%) |  |  |  |  |
| LSA |  |  |  |  |  | 0.062 | 1 | 0.760 | 1 |
| 0 | 7(13.0%) | 39(37.1%) | 84(37.3%) | 4(17.4%) | 3(9.7%) |  |  |  |  |
| 1 | 37（68.5%） | 58(55.2%) | 123(54.7%) | 14(60.9%) | 20(64.5%) |  |  |  |  |
| 2(Positive) | 10（18.5%） | 8(7.6%) | 18(8.0%) | 5(21.7%) | 8(25.8%) |  |  |  |  |
| TTA |  |  |  |  |  | 0.428 | 0.511 | 0.513 | 1 |
| 0 | 11(20.4%) | 60(57.1%) | 81(36.0%) | 12(52.2%) | 7(22.6%) |  |  |  |  |
| 1 | 35(64.8%) | 35(33.3%) | 128(56.9%) | 6(26.1%) | 18(58.1%) |  |  |  |  |
| 2(Positive) | 8(14.8%) | 10(9.5%) | 16(7.1%) | 5(21.7%) | 6(19.4%) |  |  |  |  |
| TPA |  |  |  |  |  | 0.774 | 0.848 | 1 | 1 |
| 0 | 21(38.9%) | 60(57.1%) | 122(54.2%) | 9(39.1%) | 16(51.6%) |  |  |  |  |
| 1 | 29(53.7%) | 35(33.3%) | 78(34.7%) | 12(52.2%) | 11(35.5%) |  |  |  |  |
| 2(Positive) | 4(7.4%) | 10(9.5%) | 25(11.1%) | 2(8.7%) | 4(12.9%) |  |  |  |  |
| AChA |  |  |  |  |  | 0.064 | 1 | 0.607 | 0.166 |
| 0 | 12(22.2%) | 54(51.4%) | 12(22.2%) | 9(39.1%) | 4(12.9%) |  |  |  |  |
| 1 | 21(38.9%) | 26(24.8%) | 21(38.9%) | 7(30.4%) | 11(35.5%) |  |  |  |  |
| 2(Positive) | 21(38.9%) | 25(23.8%) | 21(38.9%) | 7(30.4%) | 16(51.6%) |  |  |  |  |
| PChA |  |  |  |  |  | 0.824 | 0.495 | 0.542 | 1 |
| 0 | 22(40.7%) | 76(72.4%) | 150(66.7%) | 15(65.2%) | 20(64.5%) |  |  |  |  |
| 1 | 22(40.7%) | 12(11.4%) | 46(20.4%) | 2(8.7%) | 4(12.9%) |  |  |  |  |
| 2(Positive) | 10(18.5%) | 17(16.2%) | 29(12.9%) | 6(26.1%) | 7(22.6%) |  |  |  |  |
| PCA involvement | 12(22.2%) | 31(29.5%) | 88(39.1%) | 11(47.8%) | 16(51.6%) | 0.353 | 0.110 | 0.032* | 1 |

LSA, lenticulostriate artery; TTA, thalamotuberal artery; TPA, thalamoperforating artery; AChA, anterior choroidal artery; PChA, posterior choroidal arteries; PCA, posterior cerebral artery; P1 value indicates aa-hemispheres group vs ai-hemispheres group; P2 value indicates ai-hemispheres group vs ii-hemispheres group; P3 value indicates aa-hemispheres group vs ah-hemispheres group; P4 value indicates ah-hemispheres group vs hh-hemispheres group. *p < 0.05.
